# Drug-drug interaction assessment based on a large-scale spontaneous reporting system for hepato- and renal-toxicity, and thrombocytopenia with concomitant low-dose methotrexate and analgesics use

**DOI:** 10.1186/s40360-024-00738-6

**Published:** 2024-02-01

**Authors:** Takeshi Honma, Kenji Onda, Koichi Masuyama

**Affiliations:** 1 Bohsei Pharmacy, Isehara, Kanagawa Japan; 2 Department of Clinical Pharmacology, School of Pharmacy, Tokyo University of Pharmacy and Life Sciences, Tokyo, Japan; 3 Regulatory Science laboratory, School of Pharmacy, Tokyo University of Pharmacy and Life Sciences, Tokyo, Japan

**Keywords:** FAERS, Rheumatoid arthritis, Methotrexate, NSAIDs, Acetaminophen, Drug-drug interaction, Hepatotoxicity, Renaltoxicity, Thrombocytopenia

## Abstract

**Background:**

Methotrexate (MTX) is the cornerstone of rheumatoid arthritis (RA) treatment and is highly effective with low-dose intermittent administration. MTX is occasionally used in combination with non-steroidal anti-inflammatory drugs (NSAIDs) and acetaminophen (APAP)/paracetamol for pain or inflammation control. With MTX treatment, the side effects, such as hepatotoxicity, renal failure, and myelosuppression should be considered. These are also seen with analgesics treatment.

**Methods:**

We used a large spontaneously reported adverse event database (FAERS [JAPIC AERS]) to analyze whether the reporting of adverse events increased upon MTX and analgesic therapy in patients with RA.

**Results:**

After identifying RA cases, the crude reporting odds ratios (cRORs) for hepatotoxicity, renal failure, and thrombocytopenia associated with the use of MTX, APAP, or NSAIDs were calculated by disproportionality analysis, which revealed significantly higher cRORs for these events. No analgesics showed consistent positive signals for drug-drug interaction (DDI) with concomitant low-dose MTX analyzed using four algorithms for DDI interaction (the Ω shrinkage measure, additive or multiplicative, and combination risk ratio models). However, in renal failure and thrombocytopenia, loxoprofen (Ω_025_ = 0.08) and piroxicam (Ω_025_ = 0.46), and ibuprofen (Ω_025_ = 0.74) and ketorolac (Ω_025_ = 3.52), respectively, showed positive signals in the Ω shrinkage measure model, and no consistency was found among adverse events or NSAIDs.

**Conclusions:**

Studies using spontaneous reporting systems have limitations such as reporting bias or lack of patient background; however, the results of our comprehensive analysis support the results of previous clinical or epidemiological studies. This study also demonstrated the usefulness of FAERS for DDI assessment.

**Supplementary Information:**

The online version contains supplementary material available at 10.1186/s40360-024-00738-6.

## Background

High methotrexate (MTX) doses have been used to treat hematological malignancies and sarcomas. In contrast, low-dose intermittent MTX is highly efficacious for rheumatoid arthritis (RA) and psoriasis [1]. In both cases, side effects including gastrointestinal disorders, hepato- and renal toxicity, interstitial lung disease, myelosuppression, and infection must be considered [2, 3]. Prevention, early detection, and management of these side effects are necessary to ensure safe MTX therapy.

MTX is mainly excreted from the kidneys; thus, there is an increased risk of myelosuppression, a dose-dependent side effect, in cases of renal dysfunction [4]. MTX dose adjustment is necessary for patients with renal impairment. MTX treatment should be avoided in patients with severe renal dysfunction, including hemodialysis [5, 6].

Low-dose MTX is used in combination with biological agents, such as tumor necrosis factor-alpha inhibitors and disease-modifying antirheumatic drugs (DMARDs). Nonsteroidal anti-inflammatory drugs (NSAIDs) or acetaminophen (APAP)/paracetamol are often used to treat pain and control inflammation. NSAIDs suppress prostaglandin synthesis via cyclooxygenase inhibition and exert antipyretic, analgesic, and anti-inflammatory effect [7]. The common side effects of NSAIDs include gastrointestinal disorders, renal failure, and cardiac toxicity. Urinary albumin or serum creatinine monitoring is required in patients with impaired renal function. APAP has also been used to control pain in patients with RA. Unlike NSAIDs, APAP has been considered to have limited anti-inflammatory effect, but a typical adverse effect is hepatotoxicity [8].

Previous studies on MTX drug-drug interactions (DDI) have shown that various medicines can enhance the effects of MTX, especially at high MTX doses [9, 10]. NSAIDs have pharmacokinetic interactions with high-dose MTX. Most MTX is excreted from the renal glomerulus in an unchanged form [11], mediated by the uptake or excretion of pharmacological transporters, including OAT1, OAT3, MRP4 [9, 12]. NSAIDs also decrease MTX renal excretion by inhibiting kidney prostaglandin production [13], thereby decreasing renal blood flow. NSAIDs are the substrates of these transporters [12, 14]. Conversely, the interaction between NSAIDs and low-dose MTX is less clinically significant. However, an increased risk of MTX-related side effects due to concomitant low-dose MTX and NSAIDs has been reported [15, 16]. A Cochrane review mentioned that the combined use of NSAIDs and low-dose MTX leads to transient thrombocytopenia, and the combination of low-dose MTX and an anti-inflammatory dose of aspirin should be avoided [17].

APAP is a commonly used analgesic that causes hepatotoxicity, which is a typical side effect of MTX. APAP overdose can trigger toxic liver injury; however, a clinical dose of APAP is rarely harmful. However, there are some cases of liver injury in cases that have risk factors, including chronic alcohol consumption [18–21]. A Cochrane review of the concomitant use of low-dose MTX and analgesics did not include studies on side effects when low-dose MTX was used concomitantly with APAP [17, 22]. Therefore, quantitative information regarding the safety of concomitant low-dose MTX and APAP, including hepatotoxicity, is lacking.

The US Food and Drug Administration’s (FDA) Adverse Event Reporting System (FAERS) is a spontaneous adverse reporting system that gathers reports on drug use and adverse events [23]. Using FAERS, one of the world’s largest spontaneous reporting databases, is helpful for analyzing infrequent adverse events in the post-marketing phase, and various methods to detect signals due to drug-induced adverse events have been proposed and used for pharmacovigilance practice [24]. FAERS is also used to assess potential DDIs based on adverse event reports with concomitant drug use or prophylactic drug identification that mitigate drug-induced adverse events [25–27]. Owing to the nature of the spontaneous reporting system, there are some limitations, such as the lack of ability to aggregate real frequency of side effects and reporting biases. However, FAERS poses some advantages; it contains large data sources based on clinical situations and the comprehensiveness of medications and adverse events. Various studies have been conducted based on these characteristics.

For DDI analysis using spontaneous reporting systems, a comparison of adjusted reporting odds ratio (aROR) calculated via logistic regression analysis under certain conditions has been proposed [28, 29]. Additionally, the Ω shrinkage measure model [30], additive and multiplicative models [31], and combination risk ratio model [32] have been proposed to detect DDI signals between two drugs. Although no standard method has been established, the Ω shrinkage measure model is reported to be the most conservative [33].

To date, there has been no comprehensive report analyzing the aspect of the large spontaneous reporting system originating from clinical situations and whether the trend of each adverse event differs when low-dose MTX is concomitantly used with NSAIDs or APAP. Such an analysis could provide important supplemental information for safe MTX therapy. Here, we conducted a DDI analyses based on the number of reports in FAERS, the world’s largest spontaneous reporting system, regarding low-dose MTX and concomitant analgesics in patients with RA.

First, RA cases treated with low-dose MTX were extracted from all reported cases in FAERS, and the crude ROR (cROR) for hepatotoxicity, renal failure, and thrombocytopenia were tabulated. Next, we calculated the four evaluation indices of DDI in spontaneous reporting systems to determine whether the concomitant use of MTX and NSAID or APAP would increase reporting signals on MTX-related adverse events in patients with RA. Based on this analysis, we investigated whether the concomitant use of low-dose MTX and NSAIDs or APAP influences MTX-related adverse event occurrence and compared them with previous epidemiological studies.

## Methods

### Data source and mining

The analysis was performed using JAPIC AERS (Japan Pharmaceutical Information Center) based on FAERS data from the 4th quarter of 1997 to the 1st quarter of 2019. JAPIC AERS has undergone data cleaning; therefore, duplicate reports of the same patient have been deleted. As FAERS is an anonymized public database, it was exempt from institutional review board approval. The FAERS database contains seven data tables; we used the DRUG (drug), REAC (adverse event), and INDI (indication) tables. Each table was connected using PrimaryID and analyzed using a relational database software (Microsoft Access 2016).

### Definition of adverse events

All adverse event reports were extracted using the Preferred Term (PT) described in the Medical Dictionary for Regulatory Activities (MedDRA) ver.22.0. Hepatotoxicity was defined using the PTs included in the Standard MedDRA Queries (SMQ) 20000006. One type of renal failure was defined as renal failure 1 (RF1) using the SMQ code 20000003. To assess the different aspects of renal toxicity, other definitions of renal failure were adopted (Hamano et al., 2021) and renal failure 2 (RF2) was defined in this study. Thrombocytopenia was defined using SMQ code 20000031. All PT lists are described in the Supplementary Information.

### Disproportionality analysis

**Fig. 1:**
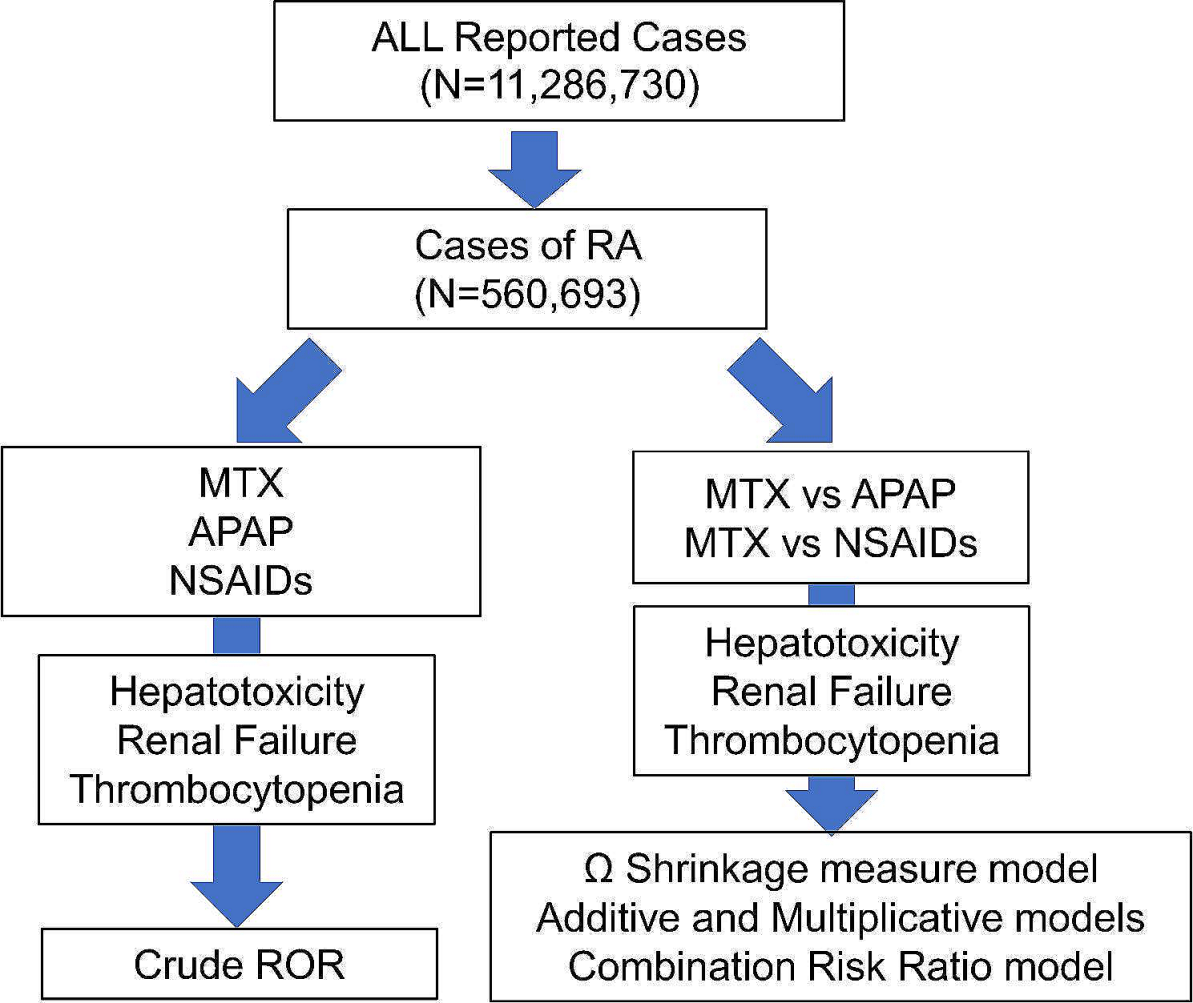
Flow chart for determining crude reporting odds ratios (cRORs) of methotrexate (MTX)-related adverse events and drug-drug interactions between MTX and analgesics. A disproportionality analysis was performed focusing on MTX use and respective adverse events such as hepatotoxicity, renal failure, and thrombocytopenia in cases of rheumatoid arthritis (RA) patients APAP: acetaminophen; NSAIDs: non-steroidal anti-inflammatory drugs

**Fig. 2:**
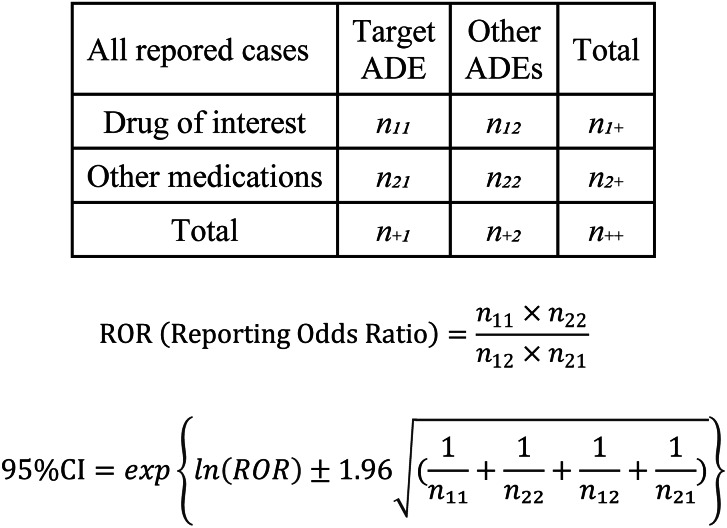
Crude reporting odds ratios (cRORs) calculation, 95% confidence intervals (95% CIs) values for each adverse drug event based on 2 × 2 contingency tables. cRORs for respective adverse events were calculated with the 2 × 2 contingency table among cases of rheumatoid arthritis (RA) patients ADE: adverse drug event

The analysis was performed according to the flowchart shown in Fig. 1. First, RA cases were extracted from all reported cases using the INDI table, and a disproportionality analysis was performed focusing on each adverse event (hepatotoxicity, RF1, RF2, and thrombocytopenia). We created 2 × 2 contingency tables based on the number of cases in which MTX and analgesics (NSAIDs and APAP) were used and the number of cases in which related adverse events were reported, and the reported odds ratio (ROR), 95% confidence intervals (CI), and χ^2^ values were calculated (Fig. 2).

### Signal detection of adverse events in concomitant use of MTX and analgesics in RA patients

**Fig. 3:**
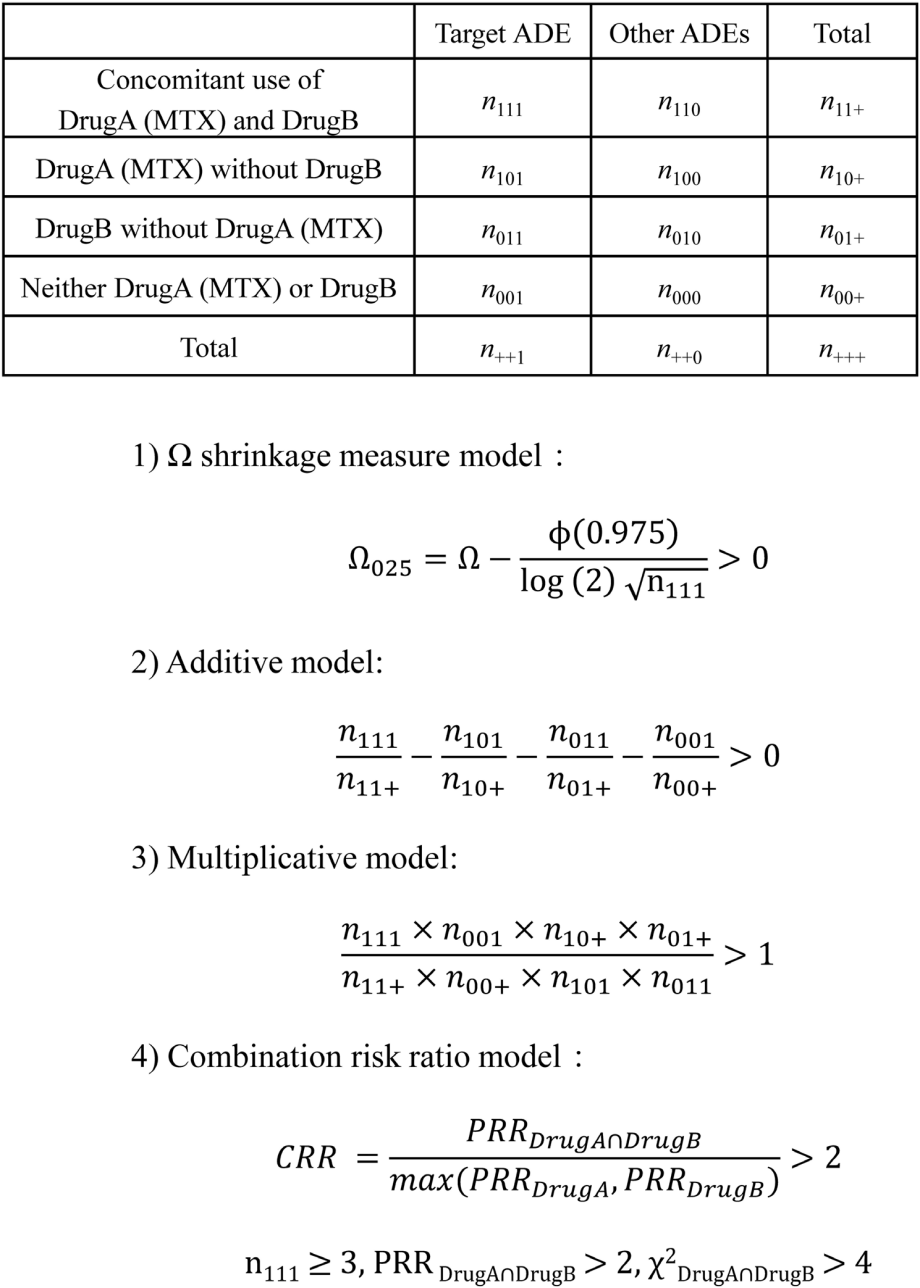
Drug-drug interaction signal analysis based on the number of reported cases of rheumatoid arthritis (RA) patients assigned to 4 × 2 contingency tables. The four drug-drug interaction algorithms were described in the materials and method section. MTX: methotrexate; ADE: adverse drug events

Next, using the extracted cases of patients with RA from the INDI table, we created 4 × 2 contingency tables regarding the presence or absence of each adverse event reported when MTX was combined with each analgesic in the RA cases (Fig. 3). Four evaluation criteria for the DDI analysis were adopted: (1) Ω shrinkage measure [30], (2) additive, (3) multiplicative [31], and (4) combination risk ratio models [32]. Data mining and statistical analyses were performed using Microsoft Access 2016 (Microsoft Co., Ltd., Tokyo, Japan). We independently developed the aggregation system using Microsoft Access and confirmed that the calculation of these indices yielded consistent results compared to previous studies [34, 35].

## Results

### Crude odds ratio (cROR) for each adverse event (hepatotoxicity, RF1, RF2, and thrombocytopenia) associated with MTX and analgesic use in patients with RA

The total number of reported cases in the cleaned FAERS dataset was 11,286,730. Of these, 560,693 cases of RA were extracted from the INDI table (Fig. 1). Table 1 shows the results of the disproportionality analysis of MTX, APAP, and NSAIDs use and the adverse events of hepatotoxicity, RF1, RF2, and thrombocytopenia. The crude reported odds ratios (cROR) (95% confidence interval) for MTX were as follows: hepatotoxicity, 3.10 (3.01–3.20); RF1, 1.08 (1.02–1.15); RF2, 1.06 (1.00–1.11); and thrombocytopenia, 2.37 (2.18–2.56), all of which were significantly higher. Analysis of APAP-related adverse events, data showed hepatotoxicity, 1.80 (1.17–1.90); RF1, 1.68 (1.54–1.85); RF2, 1.71 (1.58–1.85); thrombocytopenia, 1.74 (1.52–1.99). The cROR for these adverse events were significantly high.

**Table 1:** Crude reporting odds ratio (cROR) for hepatotoxicity, renal failure, and thrombocytopenia

	Hepatotoxicity		Renal Failure 1		Renal Failure 2		Thrombocytopenia	
Drug	cROR (95%CI)	Χ^2^	cROR (95%CI)	Χ^2^	cROR (95%CI)	Χ^2^	cROR (95%CI)	Χ^2^
Methotrexate	3.1 (3.01–3.2)	5604.43	1.08 (1.02–1.15)	7.88	1.06 (1–1.11)	4.14	2.37 (2.18–2.56)	474.73
Acetaminophen	1.8 (1.71–1.9)	504.44	1.68 (1.54–1.84)	135.29	1.71 (1.58–1.85)	176.31	1.74 (1.52–1.99)	66.98
Loxoprofen	4.78 (4.2–5.45)	669.61	2.89 (2.23–3.73)	71.02	2.52 (1.96–3.23)	56.13	7.89 (6.16–10.1)	379.3
Diclofenac	2.47 (2.29–2.66)	621.61	2.1 (1.85–2.39)	133.89	2.03 (1.8–2.28)	141.9	2.25 (1.85–2.72)	72.54
Ketoprofen	3.56 (3–4.22)	243.97	2.73 (2.01–3.72)	44.61	2.12 (1.55–2.91)	22.95	3.71 (2.47–5.57)	46.33
Celecoxib	1.75 (1.62–1.88)	231.34	1.6 (1.41–1.81)	55.06	1.39 (1.23–1.57)	28.81	1.9 (1.59–2.26)	51.26
Acetylsalicylic Acid	1.67 (1.56–1.79)	226.96	1.77 (1.59–1.98)	109.51	1.91 (1.74–2.1)	181.67	1.87 (1.58–2.2)	57.89
Indometacin	2.73 (2.26–3.29)	119.37	3.46 (2.64–4.55)	90.55	3.04 (2.34–3.96)	76.05	4.56 (3.17–6.55)	80.67
Sulindac	3.43 (2.71–4.35)	118.17	1.82 (1.09–3.04)	5.45	2 (1.28–3.12)	9.69	3.19 (1.76–5.8)	16.28
Ibuprofen	1.62 (1.47–1.78)	98.41	1.39 (1.17–1.64)	14.14	1.42 (1.22–1.65)	20.24	2.07 (1.67–2.58)	45.21
Etodolac	2.39 (1.98–2.89)	87.92	1.81 (1.28–2.57)	11.39	1.86 (1.36–2.55)	15.46	1.07 (0.53–2.14)	0.04
Tiaprofenic Acid	8.01 (4.01–15.99)	49.3	1.92 (0.26–13.88)	0.43	1.57 (0.22–11.34)	0.2	4.6 (0.63–33.3)	2.76
Naproxen	1.41 (1.28–1.55)	47.49	1.31 (1.11–1.55)	9.86	1.31 (1.12–1.52)	11.94	1.69 (1.35–2.13)	20.7
Zaltoprofen	5.47 (3.04–9.85)	40.72	8 (3.7–17.3)	39.51	1.76 (0.43–7.15)	0.64	7.84 (2.48–24.81)	17.3
Rofecoxib	1.63 (1.38–1.92)	34.57	3.82 (3.18–4.59)	237.2	4.61 (3.95–5.38)	456.32	2.11 (1.46–3.05)	16.61
Meloxicam	1.36 (1.22–1.51)	29.82	1.21 (1–1.47)	3.88	1.21 (1.01–1.44)	4.47	1.27 (0.95–1.7)	2.63
Aceclofenac	3.04 (1.9–4.87)	23.85	1.74 (0.65–4.69)	1.24	2.53 (1.19–5.37)	6.25	5.26 (2.16–12.77)	16.81
Lornoxicam	3.26 (1.92–5.55)	21.4	2.37 (0.88–6.39)	3.08	2.43 (1–5.93)	4.09	5.68 (2.11–15.34)	15.04
Mefenamic Acid	2.87 (1.77–4.65)	20.15	1.74 (0.65–4.69)	1.24	1.79 (0.74–4.34)	1.7	5.26 (2.16–12.77)	16.81
Piroxicam	1.94 (1.43–2.64)	18.99	1.61 (0.93–2.79)	2.94	2.04 (1.31–3.19)	10.34	1.77 (0.79–3.95)	1.97
Oxaprozin	2.42 (1.59–3.7)	17.86	0.58 (0.14–2.31)	0.62	1.43 (0.64–3.2)	0.76	0.69 (0.1–4.9)	0.14
Flurbiprofen	2.31 (1.46–3.63)	13.8	1.92 (0.86–4.32)	2.61	2.93 (1.61–5.36)	13.49	6.21 (3.07–12.54)	33.92

Rheumatoid arthritis (RA) cases where methotrexate (MTX) or analgesics were used

The cROR fluctuated when the number of reports was small. Table 1 lists the top 20 NSAIDs with the highest number of registered cases. The cROR for the adverse event reports of hepatotoxicity during the use of all NSAIDs were significantly high. In the analysis of RF1, 13 NSAIDs showed significantly higher cROR, whereas in the case of RF2, 16 NSAIDs showed significantly higher cROR. Fifteen NSAIDs showed significantly higher cROR values for thrombocytopenia.

### Drug-drug interaction analysis of adverse event reports when MTX is combined with NSAIDs or APAP in cases of RA

DDI analyses were performed on the RA cases used in the cROR analysis, and the effects of the concomitant use of MTX and analgesics on MTX-related adverse event reports were investigated. As the cROR for each adverse event was high with APAP and most NSAIDs, the analgesics were comprehensively examined. Using the four algorithms described in the Materials and Methods, we investigated whether the combined use of MTX and analgesics could increase the signal for these adverse events.

In the hepatotoxicity analysis, 29 analgesics were reported to cause adverse events when used in combination with MTX. APAP, celecoxib, acetylsalicylic acid, and diclofenac were the most frequently reported (n_11+_) concomitant use of MTX (Table 2).

**Table 2:** Potential drug-drug interaction analysis based on adverse event reports of hepatotoxicity

MTX + Drug	n_111_	n_11+_	E_111_	Ω shrinkage model		Additive model		Multiplicative model		CRR model	
Acetaminophen	896	15,488	1235.42	-0.46 (-0.56– -0.37)	N	-0.02	N	0.43	N	0.67	N
Celecoxib	524	8,481	616.25	-0.23 (-0.36– -0.11)	N	-0.01	N	0.57	N	0.71	N
Acetylsalicylic Acid	517	9,119	695.74	-0.43 (-0.55– -0.3)	N	-0.02	N	0.47	N	0.65	N
Diclofenac	475	6,579	640.65	-0.43 (-0.56– -0.3)	N	-0.03	N	0.38	N	0.83	N
Ibuprofen	274	4,255	294.89	-0.11 (-0.28–0.06)	N	-0.01	N	0.66	N	0.74	N
Naproxen	273	5,598	387.43	-0.5 (-0.68– -0.33)	N	-0.02	N	0.51	N	0.56	N
Meloxicam	207	4,569	320.6	-0.63 (-0.83– -0.43)	N	-0.03	N	0.46	N	0.52	N
Loxoprofen	172	1,280	182.54	-0.09 (-0.3–0.13)	N	-0.01	N	0.4	N	1.06	N
Rofecoxib	74	1,265	96.27	-0.38 (-0.71– -0.05)	N	-0.02	N	0.49	N	0.66	N
Ketoprofen	68	829	121.49	-0.83 (-1.18– -0.49)	N	-0.07	N	0.24	N	0.84	N
Indometacin	66	869	96.07	-0.54 (-0.89– -0.19)	N	-0.04	N	0.33	N	0.86	N
Etodolac	61	906	92.8	-0.6 (-0.96– -0.24)	N	-0.04	N	0.33	N	0.77	N
Sulindac	36	439	61.75	-0.77 (-1.24– -0.3)	N	-0.07	N	0.25	N	0.87	N
Piroxicam	24	431	39.31	-0.7 (-1.28– -0.12)	N	-0.04	N	0.33	N	0.63	N
Oxaprozin	16	159	12.1	0.39 (-0.32–1.1)	N	0.02	P	0.85	N	1.14	N
Flurbiprofen	14	181	15.22	-0.12 (-0.87–0.64)	N	-0.01	N	0.54	N	0.88	N
Mefenamic Acid	14	129	10.09	0.45 (-0.3–1.21)	N	0.03	P	0.86	N	1.23	N
Ketorolac	10	219	14.88	-0.55 (-1.45–0.34)	N	-0.02	N	0.49	N	0.52	N
Aceclofenac	10	134	17.69	-0.79 (-1.69–0.1)	N	-0.06	N	0.25	N	0.85	N
Lornoxicam	10	93	9.55	0.06 (-0.83–0.96)	N	0	N	0.53	N	1.19	N
Tiaprofenic Acid	9	26	1.98	1.94 (0.99–2.88)	P	0.27	P	2.9	P	1.77	N
Zaltoprofen	7	55	10.68	-0.58 (-1.64–0.49)	N	-0.08	N	0.26	N	0.89	N
Acemetacin	2	46	5.09	-1.16 (-3.16–0.84)	N	-0.07	N	0.19	N	0.49	N
Dexketoprofen	2	9	0.51	1.3 (-0.7–3.3)	N	0.18	P	–	N	2.52	N
Tenoxicam	1	40	5.19	-1.92 (-4.75–0.9)	N	-0.11	N	0.09	N	0.28	N
Tolmetin	1	18	1.46	-0.39 (-3.22–2.44)	N	-0.03	N	0.41	N	0.63	N
Bromfenac	1	12	0.68	0.34 (-2.49–3.17)	N	0.05	P	–	N	0.95	N

Methotrexate (MTX) was concomitantly used with acetaminophen (APAP) or non-steroidal anti-inflammatory drugs (NSAIDs) in rheumatoid arthritis (RA) patients. n_111_, the number of adverse event cases where MTX and the analgesic were used concomitantly; n_11+_, the number of cases where MTX and the analgesic were used concomitantly; E_111_, the expected value in the Ω shrinkage measure model; CRR, combination risk ratio; P, positive signal; N, non-positive signal

In the Ω shrinkage measure model, only tiaprofenic acid (Ω_025_ = 0.99) showed an interaction signal when used in combination with MTX, whereas no interaction signal was detected with the other analgesics. In the additive model, signals (showing interaction coefficients) were detected for four drugs: oxaprozin (0.02), mefenamic acid (0.03), tiaprofenic acid (0.27), and dexketoprofen (0.18). In the multiplicative model, it was only tiaprofenic acid (0.29) as in the Ω shrinkage measure model. No analgesics were detected in the CRR Model.

RF1 interaction signals with MTX were detected in the Ω shrinkage measure model for piroxicam (Ω_025_ = 0.45), zaltoprofen (Ω_025_ = 0.5), lornoxicam (Ω_025_ = 0.16), and fenoprofen (Ω_025_ = 0.94) (Table 3).

**Table 3:** Potential drug-drug interaction analysis based on adverse event reports of renal failure 1

MTX + Drug	n_111_	n_11+_	E_111_	Ω shrinkage model		Additive model		Multiplicative model		CRR model	
Acetaminophen	266	15,488	255.28	0.06 (-0.11–0.23)	N	0	N	1.02	P	1.02	N
Celecoxib	136	8,481	143.42	-0.08 (-0.32–0.17)	N	0	N	0.92	N	0.99	N
Acetylsalicylic acid	134	9,119	192.73	-0.52 (-0.77– -0.28)	N	-0.01	N	0.66	N	0.81	N
Diclofenac	115	6,579	172.27	-0.58 (-0.84– -0.32)	N	-0.01	N	0.64	N	0.82	N
Ibuprofen	59	4,255	64.66	-0.13 (-0.5–0.24)	N	0	N	0.89	N	0.98	N
Naproxen	49	5,598	110.08	-1.16 (-1.56– -0.76)	N	-0.01	N	0.43	N	0.65	N
Meloxicam	47	4,569	72.32	-0.62 (-1.03– -0.2)	N	-0.01	N	0.63	N	0.82	N
Loxoprofen	33	1,280	44.85	-0.44 (-0.93–0.06)	N	-0.01	N	0.7	N	0.89	N
Rofecoxib	31	1,265	59.72	-0.93 (-1.44– -0.43)	N	-0.02	N	0.48	N	0.64	N
Indometacin	28	869	33.43	-0.25 (-0.79–0.28)	N	-0.01	N	0.79	N	0.93	N
Ketoprofen	19	829	28.2	-0.56 (-1.21–0.09)	N	-0.01	N	0.64	N	0.83	N
Etodolac	14	906	20.59	-0.54 (-1.3–0.22)	N	-0.01	N	0.65	N	0.83	N
Piroxicam	12	431	4.69	1.27 (0.45–2.08)	P	0.02	P	9.21	P	1.69	N
Sulindac	9	439	7.56	0.24 (-0.7–1.18)	N	0	N	1.16	P	1.1	N
Zaltoprofen	6	55	1.57	1.65 (0.5–2.8)	P	0.08	P	3.63	P	1.42	N
Ketorolac	4	219	5.82	-0.49 (-1.9–0.92)	N	-0.01	N	0.65	N	0.84	N
Flurbiprofen	4	181	3.07	0.33 (-1.08–1.75)	N	0.01	P	1.27	P	1.12	N
Lornoxicam	4	93	1.02	1.57 (0.16–2.98)	P	0.04	P	–	N	1.79	N
Fenoprofen	3	8	0.09	2.58 (0.94–4.21)	P	0.37	P	–	N	2.38	P
Mefenamic acid	2	129	2.82	-0.41 (-2.41–1.59)	N	-0.01	N	0.68	N	0.87	N
Tenoxicam	2	40	1.98	0.01 (-1.99–2.01)	N	0	N	0.95	N	1.01	N
Oxaprozin	1	159	1.74	-0.58 (-3.41–2.25)	N	0	N	1.02	P	0.56	N
Aceclofenac	1	134	4.58	-1.76 (-4.59–1.07)	N	-0.03	N	0.21	N	0.42	N
Acemetacin	1	46	0.5	0.58 (-2.25–3.41)	N	0.02	P	–	N	1.94	N
Bromfenac	1	12	0.76	0.25 (-2.58–3.08)	N	0.02	P	1.23	P	1.17	N
Suprofen	1	1	0.01	1.55 (-1.27–4.38)	N	1	P	–	N	2	N

Methotrexate (MTX) was concomitantly used with acetaminophen (APAP) or non-steroidal anti-inflammatory drugs (NSAIDs) in rheumatoid arthritis (RA) patients. n_111_, the number of adverse event cases where MTX and the analgesic were used concomitantly; n_11+_, the number of cases where MTX and the analgesic were used concomitantly; E_111_, the expected value in the Ω shrinkage measure model; CRR, combination risk ratio; P, positive signal; N, non-positive signal

In the additive model (as an interaction coefficient), eight drugs were used that gave positive signals: piroxicam (0.02), zaltoprofen (0.08), flurbiprofen (0.01), lornoxicam (0.04), fenoprofen (0.37), acemetacin (0.02), bromfenac (0.02), and suprofen (1.0). In the multiplicative model, positive signals were observed for APAP (1.02), piroxicam (9.21), sulindac (1.16), zaloprofen (3.63), flurbiprofen (1.27), oxaprozine (1.02), and bromfenac (1.23). In the CRR model, only fenoprofen (2.38) produced a positive DDI signal.

Regarding RF2 (Table 4), interaction signals with MTX were detected in the Ω shrinkage measure models for loxoprofen (Ω_025_ = 0.08), piroxicam (Ω_025_ = 0.46), and fenoprofen (Ω_025_ = 0.05). In the additive model, eight drugs, loxoprofen (0.01), piroxicam (0.02), flurbiprofen (0.01), mefenamic acid (0.02), oxaprozin (0.01), acemetacin (0.03), fenoprofen (0.25), and suprofen (1.0) showed positive DDI signals. In the multiplicative model, paracetamol (1.06), loxoprofen (1.4), piroxicam (2.37), sulindac (1.19), flurbiprofen (1.14), oxaprozin (2.1), mefenamic acid (2.8), and acemetacin (2.68) were detected as positive signals. In the CRR model, no drug was detected with a positive DDI signal.

**Table 4:** Potential drug-drug interaction analysis based on adverse event reports of renal failure 2

MTX + Drug	n_111_	n_11+_	E_111_	Ω shrinkage model		Additive model		Multiplicative model		CRR model	
Acetaminophen	329	15,488	308.93	0.09 (-0.07–0.25)	N	0	N	1.06	P	1.02	N
Acetylsalicylic acid	174	9,119	247.92	-0.51 (-0.72– -0.3)	N	-0.01	N	0.68	N	0.81	N
Celecoxib	139	8,481	159.36	-0.2 (-0.44–0.04)	N	0	N	0.86	N	0.94	N
Diclofenac	137	6,579	198.91	-0.54 (-0.78– -0.29)	N	-0.01	N	0.67	N	0.83	N
Ibuprofen	71	4,255	81.22	-0.19 (-0.53–0.14)	N	0	N	0.86	N	0.94	N
Meloxicam	60	4,569	82.83	-0.46 (-0.83– -0.1)	N	-0.01	N	0.71	N	0.87	N
Naproxen	58	5,598	134.34	-1.2 (-1.58– -0.83)	N	-0.01	N	0.42	N	0.63	N
Rofecoxib	47	1,265	84.25	-0.84 (-1.25– -0.42)	N	-0.03	N	0.53	N	0.67	N
Loxoprofen	45	1,280	31.55	0.51 (0.08–0.93)	P	0.01	P	1.4	P	1.13	N
Indometacin	33	869	32.01	0.04 (-0.45–0.54)	N	0	N	1	N	1.02	N
Etodolac	19	906	23.73	-0.31 (-0.96–0.34)	N	-0.01	N	0.78	N	0.9	N
Ketoprofen	16	829	29.29	-0.85 (-1.56– -0.15)	N	-0.02	N	0.53	N	0.73	N
Piroxicam	15	431	6.3	1.19 (0.46–1.92)	P	0.02	P	2.37	P	1.37	N
Sulindac	12	439	9.87	0.27 (-0.55–1.09)	N	0	N	1.19	P	1.1	N
Flurbiprofen	7	181	5.95	0.22 (-0.85–1.29)	N	0.01	P	1.14	P	1.07	N
Ketorolac	6	219	8.04	-0.39 (-1.55–0.76)	N	-0.01	N	0.72	N	0.87	N
Oxaprozin	4	159	2.08	0.8 (-0.61–2.22)	N	0.01	P	2.1	P	1.4	N
Mefenamic acid	4	129	1.69	1.04 (-0.37–2.45)	N	0.02	P	2.8	P	1.39	N
Aceclofenac	3	134	6.04	-0.9 (-2.53–0.73)	N	-0.02	N	0.48	N	0.72	N
Tenoxicam	3	40	2.95	0.02 (-1.61–1.65)	N	0	N	0.97	N	1.01	N
Acemetacin	2	46	0.74	1.01 (-0.99–3.01)	N	0.03	P	2.68	P	1.61	N
Fenoprofen	2	8	0.1	2.05 (0.05–4.05)	P	0.25	P	–	N	2.38	N
Lornoxicam	1	93	5.15	-1.91 (-4.74–0.91)	N	-0.04	N	0.19	N	0.36	N
Zaltoprofen	1	55	1.56	-0.46 (-3.29–2.37)	N	-0.01	N	0.62	N	0.83	N
Suprofen	1	1	0.01	1.55 (-1.28–4.38)	N	1	P	–	N	2	N

Methotrexate (MTX) was concomitantly used with acetaminophen (APAP) or non-steroidal anti-inflammatory drugs (NSAIDs) in rheumatoid arthritis (RA) patients. n_111_, the number of adverse event cases where MTX and the analgesic were used concomitantly; n_11+_, the number of cases where MTX and the analgesic were used concomitantly; E_111_, the expected value in the Ω shrinkage measure model; CRR, combination risk ratio; P, positive signal; N, non-positive signal

Finally, we analyzed thrombocytopenia (Table 5). In the Ω-shrinkage measure model, ibuprofen (Ω_025_ = 0.74) and ketorolac (Ω_025_ = 3.52) showed interaction signals with MTX. Positive signals were observed for ibuprofen (0.01), ketorolac (0.17), mefenamic acid (0.02), lornoxicam (0.01), and tenoxicam (0.02) in additive models. In the multiplicative model, signals for ibuprofen (2.08), naproxen (1.06), ketorolac (15.15), and mefenamic acid (1.25) were detected. In addition, the CRR model did not detect any signals of interaction with any analgesics.

**Table 5:** Potential drug-drug interaction analysis based on adverse event reports of thrombocytopenia

MTX + Drug	n_111_	n_11+_	E_111_	Ω shrinkage model		Additive model		Multiplicative model		CRR model	
Acetaminophen	125	15,488	166.5	-0.41 (-0.67– -0.16)	N	0	N	0.51	N	0.81	N
Acetylsalicylic acid	73	9,119	109.25	-0.58 (-0.91– -0.25)	N	0	N	0.43	N	0.8	N
Ibuprofen	67	4,255	31.32	1.08 (0.74–1.43)	P	0.01	P	2.08	P	1.57	N
Celecoxib	66	8,481	106	-0.68 (-1.03– -0.33)	N	0	N	0.39	N	0.77	N
Diclofenac	66	6,579	84.63	-0.36 (-0.7– -0.01)	N	0	N	0.49	N	1	N
Naproxen	56	5,598	46.11	0.28 (-0.1–0.66)	N	0	N	1.06	P	0.99	N
Loxoprofen	43	1,280	44.01	-0.03 (-0.46–0.4)	N	0	N	0.47	N	1.03	N
Ketorolac	39	219	2.01	3.98 (3.52–4.43)	P	0.17	P	15.15	P	1.84	N
Meloxicam	27	4,569	42.49	-0.64 (-1.19– -0.1)	N	0	N	0.5	N	0.58	N
Indometacin	20	869	16.19	0.3 (-0.34–0.93)	N	0	N	0.68	N	1.19	N
Rofecoxib	14	1,265	15.13	-0.11 (-0.86–0.65)	N	0	N	0.61	N	1.09	N
Ketoprofen	8	829	22.59	-1.44 (-2.44– -0.44)	N	-0.02	N	0.18	N	0.61	N
Sulindac	7	439	6.64	0.07 (-1–1.14)	N	0	N	0.62	N	1.17	N
Etodolac	4	906	8.29	-0.97 (-2.38–0.45)	N	0	N	0.38	N	0.43	N
Piroxicam	4	431	4.25	-0.08 (-1.49–1.34)	N	0	N	0.7	N	0.91	N
Mefenamic acid	4	129	1.9	0.91 (-0.51–2.32)	N	0.02	P	1.25	P	1.39	N
Flurbiprofen	3	181	8.02	-1.28 (-2.92–0.35)	N	-0.03	N	0.17	N	0.63	N
Lornoxicam	3	93	1.66	0.69 (-0.94–2.33)	N	0.01	P	1	N	1.34	N
Oxaprozin	1	159	1.18	-0.16 (-2.99–2.66)	N	0	N	–	N	0.62	N
Aceclofenac	1	134	6.48	-2.22 (-5.05–0.61)	N	-0.04	N	0.07	N	0.33	N
Zaltoprofen	1	55	3.27	-1.33 (-4.16–1.5)	N	-0.04	N	0.14	N	0.55	N
Tenoxicam	1	40	0.3	0.91 (-1.92–3.74)	N	0.02	P	–	N	2.03	N

Methotrexate (MTX) was concomitantly used with acetaminophen (APAP) or non-steroidal anti-inflammatory drugs (NSAIDs) in rheumatoid arthritis (RA) patients. n_111_, the number of adverse event cases where MTX and the analgesic were used concomitantly; n_11+_, the number of cases where MTX and the analgesic were used concomitantly; E_111_, the expected value in the Ω shrinkage measure model; CRR, combination risk ratio; P, positive signal; N, non-positive signal

## Discussion

We investigated typical MTX-related adverse effects, such as hepato- and renal toxicity and thrombocytopenia, in combination with analgesics using FAERS, one of the world’s largest spontaneous report databases.

Reported cases of patients with RA were selected from all FAERS datasets, and the cROR was calculated using univariate analysis with disproportionality analysis for MTX and analgesic use. Trends in positive signals for these adverse events were confirmed. We then analyzed the DDI signals between MTX and the analgesics using four evaluation indices.

Different methods for detecting DDI signals between two drugs in spontaneous report databases have been proposed (the Ω shrinkage measure, additive, multiplicative, and combination risk ratio models). One study comparing these models showed that the Ω shrinkage measure model is the most conservative [33]. In addition, additive and multiplicative models tend to detect signals when the number of cases is small, and there is no *de facto* standard method. Using these four models, we conducted a comprehensive interaction analysis of the analgesics used in combination with MTX. According to our results, the Ω shrinkage measure and combination risk ratio models tended to detect fewer interaction signals. In the additive model and the multiplicative model, there was a tendency for positive signals to be observed for analgesics with few reports of combined use with MTX.

Low-dose MTX therapy can cause hepatotoxicity, renal failure, and myelosuppression. These side effects are in common with those of analgesics. In this study, we conducted a univariate analysis of MTX and analgesics for hepatotoxicity, renal failure, and thrombocytopenia, and found that MTX or APAP showed significantly higher cROR for all these adverse events. We also confirmed that many NSAIDs exhibited significantly high cROR. Since cROR analysis does not exclude the effects of confounding factors, such as concomitant medications, it is inappropriate to simply compare the cROR values. However, the overall tendency of each analgesic, suggested that there was an increased risk of adverse events.

Next, we analyzed the effects of concomitant use of MTX with APAP or NSAIDs on adverse event reporting, focusing on patients with RA, using the Ω shrinkage measure, additive, multiplicative, and combination risk ratio models. In the hepatotoxicity analysis, no positive signal was detected by any analysis algorithm for the concomitant use of MTX and APAP, suggesting that combined use does not increase the risk of hepatotoxicity. In addition, in the Ω shrinkage measure model analysis, all NSAIDs (except tiaprofenic acid) showed no increase in hepatotoxicity when used in combination with MTX. No NSAIDs showed a consistent positive signal among the four algorithms, indicating that none of the analgesics clearly increased MTX-associated hepatotoxicity.

In the DDI analysis of renal failure 1 between MTX and analgesics, positive signals were detected in three of the four analytical methods for piroxicam, zaltoprofen, and fenoprofen. Combination risk ratio model analysis detected a positive signal only for fenoprofen. The number of reported cases in which NSAIDs and MTX were used in combination is small, and univariate analysis did not detect a positive signal for piroxicam and fenoprofen in renal failure 1. Further investigation of this relationship is required. No positive signal was detected in any of the analytical methods when MTX and APAP were used in combination.

In the case of the analysis of renal failure 2, three indices, including Ω shrinkage measure, showed interaction signals for MTX and concomitant loxoprofen or piroxicam, but the combination risk ratio model did not detect interaction signals for any of the drugs. The fact that the number of cases of concomitant loxoprofen or piroxicam plus MTX (n_11+_) was not small (1,280 and 431, respectively) and the detection of positive signals in the three algorithms, including the Ω shrinkage model, suggests the existence of an interaction signal with MTX. The additive and multiplicative models detected slightly positive signals for some NSAIDs that were used less frequently with MTX. These positive signals disappeared during the Ω shrinkage measure.

A 2012 Cochrane review indicated that low-dose MTX should be used with caution due to hepatotoxicity and renal dysfunction when used in combination with anti-inflammatory doses of aspirin [17]. Furthermore, MTX used at low doses (≤ 30 mg) does not affect concomitant use with NSAIDs. We previously confirmed that the maximum weekly dose of MTX in RA cases is mostly distributed at no more than 25 mg, using information registered in FAERS [29]. However, the doses of analgesics, including aspirin, were not investigated in our FAERS data because of difficulties in the analysis. Further investigations are required to determine whether high-dose aspirin affects MTX-related adverse events.

In thrombocytopenia, ibuprofen and ketorolac showed an interaction with MTX by the Ω shrinkage measure, the additive, and the multiplicative models. In the combination risk ratio model, no signal of interaction with MTX was observed for any analgesic. A Cochrane review reported that the combination of low-dose MTX and NSAIDs resulted in a brief and mild increase in thrombocytopenia [17, 36]. APAP and most other NSAIDs did not interact with MTX. Given that a number of cases (n_11+_) used concomitant MTX and ibuprofen (4,255) or ketorolac (219), this suggests that more attention is necessary than for other NSAIDs when used concomitantly with MTX.

This study had several limitations. As the FAERS consists of spontaneously reported data, it should be noted that there are various reporting biases, and the true frequency of adverse events cannot be evaluated.

A competition bias potentially arises when a particular drug is associated with numerous adverse event reports, thereby diminishing the signal for other adverse events, and vice versa [37]. This bias is recognized in disproportionality analyses and may be relevant in the context of DDI analyses that involve expanded contingency tables.

The algorithms proposed for analysis in this study assumed interactions between two drugs and did not consider combinations of three or more drugs [38]. The influence of potential confounding factors, such as other concomitant drugs (including DMARDs and biologics), cannot be excluded. Age, sex, disease background (hepatic and renal function), information on dosage (e.g., aspirin), dosage form, administration frequency, route, and lifestyle (e.g., alcohol intake) were not considered. Therefore, the findings of this study should be interpreted as hypothetical and do not necessarily reflect the actual situation. Further investigation, including the utilization of other large-scale databases, may be required to validate these hypotheses. Based on these points, the background factors of individual cases should be considered in actual clinical pharmacotherapy settings, and MTX and analgesics should be used with caution in patients with renal and hepatic impairment. These adverse events can occur even when MTX and analgesics are not administered concomitantly. Despite these limitations, a comprehensive analysis using the world’s largest data source is of significance. Such information could complement meta-analytical studies, which may be subject to research biases and heterogeneity.

## Conclusions

To the best of our knowledge, this is the first study to use the FAERS to analyze the effects of concomitant analgesics on various MTX-related adverse events in patients with RA. The concomitant use of APAP or NSAIDs in low-dose MTX therapy is unlikely to lead to an overall increase in adverse events, such as hepatotoxicity. A few NSAIDs were associated with an increased reporting of renal failure or thrombocytopenia; however, NSAIDs as a whole appeared to have little influence. These results support previous epidemiological studies showing that the effects of concomitant use of low-dose MTX and analgesics are minor. The study also demonstrated that FAERS analysis was useful for evaluating adverse drug interactions.

### Electronic supplementary material

Below is the link to the electronic supplementary material.

Supplementary Material 1

## Data Availability

Publicly available datasets were analyzed in this study. This data can be found here: https://www.fda.gov/drugs/questions-and-answers-fdas-adverse-event-reporting-system-faers/fda-adverse-event-reporting-system-faers-public-dashboard.
