# Supplementary material for: Drug-drug interaction assessment based on a large-scale spontaneous reporting system for hepato- and renal-toxicity, and thrombocytopenia with concomitant low-dose methotrexate and analgesics use
Source: BMC Pharmacol Toxicol. 2024 Feb 1;25:13. doi: 10.1186/s40360-024-00738-6 (PMC10832291; doi:10.1186/s40360-024-00738-6)
Supplement: Supplementary file 1 — Supplementary Material 1 [file 40360_2024_738_MOESM1_ESM.docx]

Supplementary Information

Supplementary Table 1 Complete lists of the preferred terms used for definition of adverse events.

## PT code for hepatotoxicity

| Ptcode | PT term |
| --- | --- |
| 10000028 | 5'nucleotidase increased |
| 10000804 | Acute hepatic failure |
| 10001547 | Alanine aminotransferase abnormal |
| 10001551 | Alanine aminotransferase increased |
| 10001942 | Ammonia abnormal |
| 10001946 | Ammonia increased |
| 10003445 | Ascites |
| 10003477 | Aspartate aminotransferase abnormal |
| 10003481 | Aspartate aminotransferase increased |
| 10003547 | Asterixis |
| 10003827 | Autoimmune hepatitis |
| 10004269 | Benign hepatic neoplasm |
| 10004659 | Biliary cirrhosis |
| 10004664 | Biliary fibrosis |
| 10004685 | Bilirubin conjugated increased |
| 10004792 | Biopsy liver abnormal |
| 10005364 | Blood bilirubin increased |
| 10005370 | Blood bilirubin unconjugated increased |
| 10005429 | Blood cholinesterase abnormal |
| 10005430 | Blood cholinesterase decreased |
| 10005518 | Blood fibrinogen abnormal |
| 10005520 | Blood fibrinogen decreased |
| 10005818 | Blood thrombin abnormal |
| 10005820 | Blood thrombin decreased |
| 10005824 | Blood thromboplastin abnormal |
| 10005826 | Blood thromboplastin decreased |
| 10006408 | Bromosulphthalein test abnormal |
| 10008635 | Cholestasis |
| 10008909 | Chronic hepatitis |
| 10009736 | Coagulation factor decreased |
| 10009746 | Coagulation factor IX level decreased |
| 10009754 | Coagulation factor V level decreased |
| 10009761 | Coagulation factor VII level decreased |
| 10009775 | Coagulation factor X level decreased |
| 10010075 | Coma hepatic |
| 10010186 | Complications of transplanted liver |
| 10017688 | Gamma-glutamyltransferase abnormal |
| 10017693 | Gamma-glutamyltransferase increased |
| 10018704 | Granulomatous liver disease |
| 10018821 | Haemangioma of liver |
| 10019621 | Hepaplastin abnormal |
| 10019622 | Hepaplastin decreased |
| 10019629 | Hepatic adenoma |
| 10019637 | Hepatic atrophy |
| 10019641 | Hepatic cirrhosis |
| 10019646 | Hepatic cyst |
| 10019660 | Hepatic encephalopathy |
| 10019663 | Hepatic failure |
| 10019668 | Hepatic fibrosis |
| 10019670 | Hepatic function abnormal |
| 10019692 | Hepatic necrosis |
| 10019695 | Hepatic neoplasm |
| 10019705 | Hepatic pain |
| 10019708 | Hepatic steatosis |
| 10019717 | Hepatitis |
| 10019727 | Hepatitis acute |
| 10019754 | Hepatitis cholestatic |
| 10019755 | Hepatitis chronic active |
| 10019759 | Hepatitis chronic persistent |
| 10019772 | Hepatitis fulminant |
| 10019795 | Hepatitis toxic |
| 10019823 | Hepatoblastoma recurrent |
| 10019837 | Hepatocellular injury |
| 10019842 | Hepatomegaly |
| 10019845 | Hepatorenal failure |
| 10019846 | Hepatorenal syndrome |
| 10019847 | Hepatosplenomegaly |
| 10019851 | Hepatotoxicity |
| 10020575 | Hyperammonaemia |
| 10020578 | Hyperbilirubinaemia |
| 10020942 | Hypoalbuminaemia |
| 10020973 | Hypocoagulable state |
| 10021085 | Hypoprothrombinaemia |
| 10021209 | Icterus index increased |
| 10022592 | International normalised ratio abnormal |
| 10022595 | International normalised ratio increased |
| 10023025 | Ischaemic hepatitis |
| 10023126 | Jaundice |
| 10023129 | Jaundice cholestatic |
| 10023136 | Jaundice hepatocellular |
| 10023321 | Kayser-Fleischer ring |
| 10024275 | Leucine aminopeptidase increased |
| 10024670 | Liver disorder |
| 10024690 | Liver function test abnormal |
| 10024712 | Liver tenderness |
| 10024714 | Liver transplant |
| 10024715 | Liver transplant rejection |
| 10025129 | Lupoid hepatic cirrhosis |
| 10027761 | Mixed hepatocellular cholangiocarcinoma |
| 10030210 | Oesophageal varices haemorrhage |
| 10036200 | Portal hypertension |
| 10036204 | Portal shunt |
| 10037005 | Protein C decreased |
| 10037048 | Prothrombin level abnormal |
| 10037050 | Prothrombin level decreased |
| 10037057 | Prothrombin time abnormal |
| 10037063 | Prothrombin time prolonged |
| 10037068 | Prothrombin time ratio increased |
| 10039012 | Reye's syndrome |
| 10041519 | Spider naevus |
| 10041661 | Splenorenal shunt |
| 10045428 | Ultrasound liver abnormal |
| 10048245 | Yellow skin |
| 10048611 | Cholaemia |
| 10049483 | Glutamate dehydrogenase increased |
| 10049547 | Antithrombin III decreased |
| 10049631 | Oedema due to hepatic disease |
| 10050792 | Urine bilirubin increased |
| 10050842 | Liver carcinoma ruptured |
| 10050897 | Portal hypertensive gastropathy |
| 10051010 | Duodenal varices |
| 10051012 | Gastric varices |
| 10051015 | Radiation hepatitis |
| 10051081 | Nodular regenerative hyperplasia |
| 10051120 | Protein S decreased |
| 10051125 | Hypofibrinogenaemia |
| 10051319 | Thrombin time abnormal |
| 10051343 | Bile output decreased |
| 10051344 | Bile output abnormal |
| 10051390 | Thrombin time prolonged |
| 10051603 | Liver and pancreas transplant rejection |
| 10051736 | Protein S abnormal |
| 10051924 | Hypercholia |
| 10052274 | Hepatopulmonary syndrome |
| 10052279 | Renal and liver transplant |
| 10052285 | Focal nodular hyperplasia |
| 10052550 | Liver induration |
| 10052554 | Foetor hepaticus |
| 10052716 | Peritoneovenous shunt |
| 10053219 | Non-alcoholic steatohepatitis |
| 10053244 | Hepatocellular foamy cell syndrome |
| 10053973 | Hepatic cyst ruptured |
| 10054125 | Perihepatic discomfort |
| 10054885 | Hepatic haemangioma rupture |
| 10054889 | Transaminases increased |
| 10055110 | Hepatic cancer metastatic |
| 10056091 | Varices oesophageal |
| 10056536 | X-ray hepatobiliary abnormal |
| 10056956 | Subacute hepatic failure |
| 10057110 | Hepatic mass |
| 10057572 | Gastric varices haemorrhage |
| 10057573 | Chronic hepatic failure |
| 10058117 | Ocular icterus |
| 10058477 | Blood bilirubin abnormal |
| 10058517 | Hypothrombinaemia |
| 10059318 | Hepatic cancer stage I |
| 10059319 | Hepatic cancer stage II |
| 10059324 | Hepatic cancer stage III |
| 10059325 | Hepatic cancer stage IV |
| 10059570 | Blood alkaline phosphatase increased |
| 10059571 | Blood alkaline phosphatase abnormal |
| 10059766 | Haemorrhagic ascites |
| 10060794 | Hepatic enzyme decreased |
| 10060795 | Hepatic enzyme increased |
| 10061009 | Bilirubin excretion disorder |
| 10061203 | Hepatobiliary neoplasm |
| 10061770 | Coagulation factor IX level abnormal |
| 10061771 | Coagulation factor V level abnormal |
| 10061772 | Coagulation factor VII level abnormal |
| 10061774 | Coagulation factor X level abnormal |
| 10061918 | Prothrombin time ratio abnormal |
| 10061947 | Liver scan abnormal |
| 10061997 | Hepatectomy |
| 10061998 | Hepatic lesion |
| 10062000 | Hepatobiliary disease |
| 10062001 | Hepatoblastoma |
| 10062040 | Liver operation |
| 10062685 | Hepatic enzyme abnormal |
| 10062688 | Transaminases abnormal |
| 10063075 | Cryptogenic cirrhosis |
| 10064190 | Cholestatic pruritus |
| 10064558 | Total bile acids increased |
| 10064668 | Hepatic infiltration eosinophilic |
| 10064676 | Graft versus host disease in liver |
| 10064712 | Mitochondrial aspartate aminotransferase increased |
| 10064936 | Portal vein pressure increased |
| 10065274 | Hepatic calcification |
| 10066004 | Pneumobilia |
| 10066195 | Hepatobiliary scan abnormal |
| 10066244 | Hepatic sequestration |
| 10066263 | Acute graft versus host disease in liver |
| 10066599 | Hepatic encephalopathy prophylaxis |
| 10066758 | Mixed liver injury |
| 10066869 | Molar ratio of total branched-chain amino acid to tyrosine |
| 10067125 | Liver injury |
| 10067281 | Portopulmonary hypertension |
| 10067337 | Portal vein flow decreased |
| 10067338 | Retrograde portal vein flow |
| 10067365 | Hepatic hydrothorax |
| 10067388 | Hepatic angiosarcoma |
| 10067718 | Bilirubin conjugated abnormal |
| 10067737 | Lupus hepatitis |
| 10067796 | Haemorrhagic hepatic cyst |
| 10067823 | Splenic varices |
| 10067969 | Cholestatic liver injury |
| 10068237 | Hypertransaminasaemia |
| 10068287 | Child-Pugh-Turcotte score increased |
| 10068370 | Acquired protein S deficiency |
| 10068547 | Bacterascites |
| 10068662 | Splenic varices haemorrhage |
| 10068664 | Liver sarcoidosis |
| 10068821 | Periportal oedema |
| 10068923 | Portal hypertensive enteropathy |
| 10068924 | Anorectal varices |
| 10068925 | Anorectal varices haemorrhage |
| 10068997 | Hepatic artery flow decreased |
| 10069380 | Small-for-size liver syndrome |
| 10070479 | Urobilinogen urine increased |
| 10070953 | Reynold's syndrome |
| 10071198 | Allergic hepatitis |
| 10071265 | Diabetic hepatopathy |
| 10071502 | Intestinal varices |
| 10071634 | Deficiency of bile secretion |
| 10072160 | Chronic graft versus host disease in liver |
| 10072268 | Drug-induced liver injury |
| 10072284 | Varicose veins of abdominal wall |
| 10072319 | Gallbladder varices |
| 10072629 | Intrahepatic portal hepatic venous fistula |
| 10073069 | Hepatic cancer |
| 10073070 | Hepatic cancer recurrent |
| 10073071 | Hepatocellular carcinoma |
| 10073073 | Hepatobiliary cancer |
| 10073074 | Hepatobiliary cancer in situ |
| 10073209 | Portal vein dilatation |
| 10073215 | Peripancreatic varices |
| 10073979 | Portal vein cavernous transformation |
| 10074084 | Hepatic fibrosis marker abnormal |
| 10074150 | Biliary ascites |
| 10074151 | Parenteral nutrition associated liver disease |
| 10074352 | Liver iron concentration abnormal |
| 10074354 | Liver iron concentration increased |
| 10074413 | Hepatic fibrosis marker increased |
| 10074561 | Acquired antithrombin III deficiency |
| 10074726 | Portal fibrosis |
| 10074737 | Hyperfibrinolysis |
| 10075186 | Stomal varices |
| 10075331 | Portal tract inflammation |
| 10075895 | Liver palpable |
| 10076239 | Spontaneous intrahepatic portosystemic venous shunt |
| 10076254 | Hepatic hypertrophy |
| 10076331 | Steatohepatitis |
| 10076640 | Liver dialysis |
| 10077020 | Child-Pugh-Turcotte score abnormal |
| 10077215 | Hepatic steato-fibrosis |
| 10077259 | Non-cirrhotic portal hypertension |
| 10077281 | Splenorenal shunt procedure |
| 10077291 | Model for end stage liver disease score abnormal |
| 10077292 | Model for end stage liver disease score increased |
| 10077305 | Acute on chronic liver failure |
| 10077356 | Bilirubin urine present |
| 10077479 | Portal shunt procedure |
| 10077670 | Anti factor X activity abnormal |
| 10077671 | Anti factor X activity increased |
| 10077674 | Anti factor X activity decreased |
| 10077677 | Liver function test decreased |
| 10077692 | Liver function test increased |
| 10077861 | Cholangiosarcoma |
| 10077922 | Benign hepatobiliary neoplasm |
| 10078058 | Intestinal varices haemorrhage |
| 10078360 | Computerised tomogram liver abnormal |
| 10078962 | Immune-mediated hepatitis |
| 10079685 | Hepatic hamartoma |
| 10079686 | Hepatic lymphocytic infiltration |
| 10079889 | Hepatobiliary cyst |
| 10080429 | Primary biliary cholangitis |
| 10080824 | Glycocholic acid increased |
| 10082249 | Nonalcoholic fatty liver disease |
| 10082450 | Multivisceral transplantation |

## PT code for renal failure1

| PT | COR_PT |
| --- | --- |
| 10002847 | Anuria |
| 10003885 | Azotaemia |
| 10018875 | Haemodialysis |
| 10029155 | Nephropathy toxic |
| 10030302 | Oliguria |
| 10034660 | Peritoneal dialysis |
| 10038435 | Renal failure |
| 10038447 | Renal failure neonatal |
| 10049776 | Renal impairment neonatal |
| 10049778 | Neonatal anuria |
| 10053090 | Haemofiltration |
| 10061105 | Dialysis |
| 10062237 | Renal impairment |
| 10066338 | Continuous haemodiafiltration |
| 10069339 | Acute kidney injury |
| 10069688 | Acute phosphate nephropathy |
| 10072370 | Prerenal failure |
| 10078987 | Foetal renal impairment |

## PT code for renal failure2

| PT | COR_PT |
| --- | --- |
| 10002847 | Anuria |
| 10005462 | Blood creatine abnormal |
| 10005464 | Blood creatine increased |
| 10005481 | Blood creatinine abnormal |
| 10005483 | Blood creatinine increased |
| 10005846 | Blood urea abnormal |
| 10005851 | Blood urea increased |
| 10011372 | Creatinine renal clearance decreased |
| 10012660 | Diabetic end stage renal disease |
| 10018356 | Glomerular filtration rate abnormal |
| 10018358 | Glomerular filtration rate decreased |
| 10018932 | Haemolytic uraemic syndrome |
| 10019845 | Hepatorenal failure |
| 10019846 | Hepatorenal syndrome |
| 10030302 | Oliguria |
| 10038428 | Renal disorder |
| 10038435 | Renal failure |
| 10038447 | Renal failure neonatal |
| 10044501 | Traumatic anuria |
| 10049776 | Renal impairment neonatal |
| 10049778 | Neonatal anuria |
| 10056675 | Postoperative renal failure |
| 10059345 | Postrenal failure |
| 10061480 | Renal function test abnormal |
| 10062553 | Scleroderma renal crisis |
| 10063181 | Propofol infusion syndrome |
| 10063431 | Nail-patella syndrome |
| 10064848 | Chronic kidney disease |
| 10068230 | Cardiorenal syndrome |
| 10068447 | Creatinine renal clearance abnormal |
| 10069339 | Acute kidney injury |
| 10072370 | Prerenal failure |

## PT code for thrombocytopenia

| PT | COR_PT |
| --- | --- |
| 10027119 | Megakaryocytes decreased |
| 10035528 | Platelet count decreased |
| 10035537 | Platelet maturation arrest |
| 10035540 | Platelet production decreased |
| 10043554 | Thrombocytopenia |
| 10059440 | Platelet toxicity |
| 10076747 | Acquired amegakaryocytic thrombocytopenia |
